# Drone Applications for Emergency and Urgent Care: A Systematic Review

**DOI:** 10.1017/S1049023X22000887

**Published:** 2022-08

**Authors:** Sebastián Sanz-Martos, María Dolores López-Franco, Cristina Álvarez-García, Nani Granero-Moya, José María López-Hens, Sixto Cámara-Anguita, Pedro Luis Pancorbo-Hidalgo, Inés María Comino-Sanz

**Affiliations:** 1. Department of Nursing, University of Jaén, Spain; 2. Basic Health Area Torreperogil, District Jaén Nordeste, Servicio Andaluz de Salud, Spain; 3. Emergency Medical Drone Co-operative, Jaén, Spain

**Keywords:** drone, emergency medicine, triage, unmanned aerial vehicle

## Abstract

**Introduction:**

In recent years, the use of drones in health emergencies has increased. Among their main benefits are avoiding endangering rescuers, travelling long distances in a short time, or contacting victims in risky situations; but despite their multiple advantages, their use has not been fully demonstrated.

**Study Objective:**

This study aims to identify the available evidence on the use of drones in emergency health care compared to traditional health care.

**Methods:**

Systematic review of the literature was conducted. Search protocols were developed to locate studies that met the established selection criteria. Six experimental or quasi-experimental studies with high methodological quality published from the beginning of indexing until 2020 were included.

**Results:**

Drones covered a significantly larger area than other traditional tracking methods and were very useful for performing preliminary triage, determining needs, and knowing the scene prior to the arrival of rescuers. In addition, drones reduced the time required to locate the victim.

**Conclusions:**

Drones are an element to be taken into account when attending health emergencies as they significantly improve the distance travelled to locate accident victims, have the possibility of performing triage prior to the arrival of the health care units, and improve the time and quality of the care provided.

## Introduction

Today’s society is inconceivable without new technologies and the digital universe, and their application in the nursing profession is undoubtedly a growing and continuously advancing field.^1^ In the health care field, the use of new technologies will allow for efficient and rapid care, reducing the time required for action, which on many occasions can have a direct impact on the survival of people.^2^

Recently, the use of Unmanned Aircraft Systems (UAS), or drones, in health emergencies has increased. One of the main benefits of drones is to avoid endangering rescuers in shootings, fires, radiation, the presence of infectious agents, explosives, smoke, or gases.^3,4^ They also offer the advantage of covering long distances in a short time; they facilitate approaching places where rescuers would not be able to go, such as rural areas difficult to access or accident zones where an approach without risk to rescuers cannot be guaranteed sooner than would be possible by traditional means.^5–7^ Drones are notable for their ease of transport and flexibility of deployment as they do not require specific infrastructures.^8^

However, when assessing all their functionalities, there are some limitations regarding their use in health care. For example, they cannot carry much weight, they are challenging to use in adverse weather conditions, and their initial investment is significant, although their cost is low compared to existing standard methods such as manned aerial vehicles-helicopters. Additionally, legislation is needed to make their use safe, regulated, and controlled.^4,7,9,10^

One of the possible applications of drones in emergency care is for triaging patients in multiple-casualty accidents. Previous research by Jain, et al^11^ has compared standard practice with the use of drones in triage at a multi-casualty incident using the Simple Treatment and Rapid Transport (START) model of triage. Both groups were tested for accuracy in the first step of identifying walking (green) patients and simultaneously providing instructions through a loudspeaker, demonstrating the feasibility of triage of green casualties before the arrival of the first responders. Furthermore, although triage with a drone was 3.5 minutes slower, it could arrive 93% faster than an Emergency Medical Service, allowing remote triage before emergency services arrive and prioritizing care more effectively.

Another triage method used with drone assistance was the Sort, Assess, Life-Saving Interventions, Treatment/Transport (SALT) method where Sibley, et al^12^ used drones to initiate the first step of SALT triage (ie, general assessment of the injured). Additionally, with the images provided by the drone, they assessed the ability to identify hazards at the scene and to designate the best areas for the second step of triage and the deployment of operational areas (command post, treatment area, ambulance area, access, and evacuation routes). The results showed that 82% of the participants correctly classified 12 of the 15 victims.

Recently, Álvarez-García C, et al^13^ developed a remote triage procedure using drones. This triage procedure comprises an algorithm that assesses essential aspects such as bleeding, gait, presence of consciousness, and signs of life and subsequently classifies the injured into various priority categories. Additionally, with the help of bystanders recruited through messages broadcast through a public address system onboard the drone, this algorithm includes the possibility of indicating interventions such as compression of bleeding injuries or adaptation of the lateral safety position.

Likewise, among the possibilities for the use of drones in health care, it is worth highlighting the incorporation of instruments to transmit bio-parameters such as body temperature, heart rate, respiratory rate, and even electrocardiography or oxygen saturation so that emergency teams can prioritize care and consider in greater depth the characteristics of care that are going to be required.^7,14^

These new possibilities provided by UAS are due to the development of photo-stimulus imaging, video-analysis, and motion magnification technology, as video cameras can detect changes in facial skin imperceptible to the human eye and cardiorespiratory movements in the chest of accident victims to determine signs of life.^15,16^ Previous research has shown that the results have similar reliability values to other vital sign measurements such as pulse oximetry or a respiratory transducer belt.^15,16^

Additionally, it has been shown that the preview of the situation that the emergency team will face through thermal cameras makes it possible to prepare the rescuers better to evacuate the victims even when there is little visibility.^17^

Thus, this study identifies the available evidence on the use of drones in emergency care compared to traditional health care.

## Methods

A systematic review of the literature was conducted following the Preferred Reporting Items for Systematic Reviews and Meta-Analyses (PRISMA) statement.^18^

### Search Strategy

The search for published studies was conducted in the following bibliographic databases: Cuiden Plus (Fundación Index; Granada, Spain); Global Health (EBSCO Information Services; Ipswich, Massachusetts USA); LILACS (Latin American and Caribbean Center on Health Sciences Information; São Paulo, Brazil); Web of Science (Thomson Reuters; New York, New York USA); Scopus (Elsevier; Amsterdam, Netherlands); Cochrane (The Cochrane Collaboration; London, United Kingdom); CINAHL (EBSCO Information Services; Ipswich, Massachusetts USA); Health and Medical Complete (ProQuest; Ann Arbor, Michigan USA); IME (Spanish Society of Family and Community Medicine; Barcelona, Spain; Medline (US National Library of Medicine, National Institutes of Health; Bethesda, Maryland USA); Science Direct (Elsevier; Amsterdam, Netherlands); and Dialnet Plus (DIALNET Foundation; Universidad de La Rioja; La Rioja, Spain) with no date limit from the start of indexing of each database until March 2020 and using the terms: “drone,” “emergencies,” “uav,” “triage,” “unmanned aerial vehicle,” “drones,” “triage,” and “emergency medicine.” The individual search strings for each database can be found in Table 1. In addition, a reverse search with secondary retrieval was also performed by analyzing the bibliography of the located articles considered to be of interest.

**Table 1.** Databases and Search Strings Used in the Review

Databases	Search Strings
Cuiden Plus	Dron AND emergen
Global Health	(UAV or unmanned aerial vehicle or drones) AND (Emergency Medicine or emergency)
Lilacs	Dron$ and emergen$
IME	Dron y (emergencia o emergencias)
PubMed	(UAV[tiab] or unmanned aerial vehicle[tiab] or drones[tiab]) AND (Emergency Medicine[tiab] or emergency[tiab])
CINAHL	(AB UAV or AB unmanned aerial vehicle or AB drones) AND (AB Emergency Medicine or AB emergency)
Scopus	UAV OR unmanned AND aerial AND vehicle OR drones AND emergency AND triage
Web of Science	TI=(UAV or unmanned aerial vehicle or drones) AND TI=(Emergency Medicine or emergency)
Health and Medical Complete	ti((UAV or unmanned aerial vehicle or drones)) AND ti((Emergency Medicine or emergency))
Science Direct	(UAV or unmanned aerial vehicle or drones) AND (Emergency Medicine or emergency)
Dialnet Plus	Dron y (emergencia o emergencias)

### Study Selection Criteria

Studies were included if they met the following inclusion criteria:

- Population: Adult patients requiring care for an urgent or emergency situation.
- Intervention: Care using a UAS or drone.
- Comparator: Health care by traditional means.
- Outcome: Distance covered for locating accident victims, priority order applied to victims by triage, or effectiveness for transporting biological samples.
- Study Design: Experimental or quasi-experimental studies.

Articles in Spanish and English were included concerning the application of drones in medicine. However, it was not considered appropriate to limit the search to a specific emergency health application due to the limited information available on the subject.

Studies were selected based on their title and abstract and were obtained in full text for further analysis.

### Classification of the Results

The results of the review were grouped according to the variable studied: surface area covered by the drone, effectiveness of drones for victim location, aspects related to victim triage, or transport of samples or biological substances.

### Research Quality

The methodological quality of the included studies was assessed using the Critical Appraisal Skills Program, Spanish (CASPe) guidelines^19^ for randomized clinical trials and the Transparent Reporting of Evaluations with Nonrandomized Designs (TREND) guidelines^20^ for quasi-experimental studies. Included in the review were all experimental studies that obtained three affirmative answers to the first three questions of the CASPe guide and a score equal to or higher than eight points on the overall scale. Quasi-experimental studies were assessed for compliance with the criteria of transparency in the sample selection, treatment assignment, the analysis of potential confounding variables, extrapolability, and overall methodological robustness as a criterion of high methodological quality. The results of the evaluation of the studies are presented in Table 2 and Table 3.^11^,^21–25^

**Table 2.** Assessment of Study Quality Using the CASPe Guideline

Item	Study	
	Jain, et al^11^	Pardo-Ríos, et al^23^
1	Yes	Yes
2	Yes	Yes
3	Yes	Yes
4	No	No
5	No	Yes
6	Yes	Yes
7	Yes	No
8	No	No
9	Yes	Yes
10	Yes	Yes
11	Yes	Yes

Note: Item 1-Is the trial oriented to a clearly defined question; Item 2-Was the allocation of patients to treatments randomized; Item 3-Were all patients who entered the study adequately considered until the end of the study; Item 4-Was the blinding of patients, clinicians, and study personnel maintained; Item 5-Were the groups similar at the start of the trial; Item 6-Were the groups treated similarly at the start of the trial; Item 7-How large was the treatment effect; Item 8-What is the precision of this effect; Item 9-Can these results be applied in local setting or population; Item 10-Were all clinically important outcomes taken into account; Item 11-Do the benefits to be gained justify the risks and costs?

Abbreviation: CASPe, Critical Appraisal Skills Program Spanish.

**Table 3.** Assessment of Study Quality Using the TREND Guideline

Item	Study			
	Claesson, et al^21^	Karaka, et al^22^	Ochieng, et al^24^	Haidari, et al^25^
1	Yes	Yes	Yes	Yes
2	No	No	Yes	No
3	Yes	Yes	Yes	Yes
4	Yes	Yes	Yes	Yes
5	Yes	Yes	Yes	Yes
6	Yes	Yes	No	Yes
7	No	No	No	No
8	NA	NA	NA	NA
9	No	No	No	No
10	No	No	Yes	Yes
11	Yes	Yes	No	Yes
12	Yes	Yes	NA	No
13	No	No	NA	NA
14	No	No	NA	NA
15	No	No	NA	NA
16	Yes	Yes	Yes	Yes
17	Yes	Yes	Yes	Yes
18	No	No	No	No
19	No	No	Yes	No
20	Yes	Yes	Yes	Yes
21	Yes	Yes	No	No
22	No	Yes	Yes	Yes

Note: Item 1-Title and abstract; Item 2-Background; Item 3-Participants; Item 4-Interventions; Item 5-Objectives; Item 6-Variables; Item 7-Sample size; Item 8-Allocation method; Item 9-Blinding; Item 10-Unit of analysis; Item 11-Statistical methods employed; Item 12-Participant flow; Item 13-Recruitment; Item 14-Baseline data; Item 15-Baseline data equivalence; Item 16-Quantitative analysis; Item 17-Results and trends; Item 18-Secondary analysis; Item 19-Adverse effects found; Item 20-Interpretation; Item 21-Extrapolation; Item 22-Evidence as a whole.

Abbreviation: TREND, Transparent Reporting of Evaluations with Nonrandomized Designs.

### Extraction of Information

A form was used to collect the research design, characteristics of the participants, intervention, comparator, and main results. To ensure the quality of the process, two members of the research team conducted the literature search, and the article selection process was carried out by three members other than those who conducted the search. Thus, the included studies required the consensus of three researchers.

### Search Results

**Figure 1.**
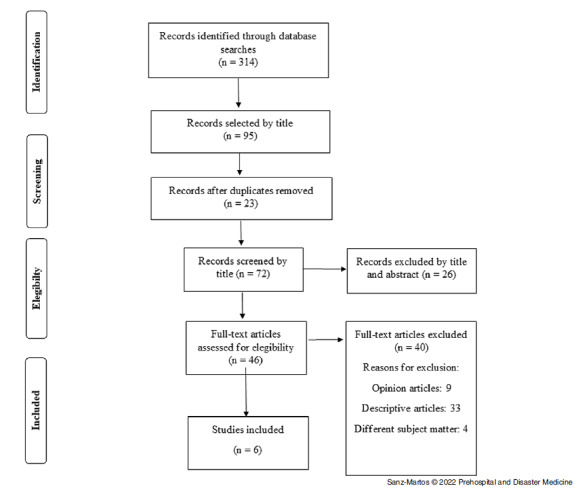
Flow Chart of the Selection Studies Adapted from the PRISMA Model.

The search strategy resulted in 314 papers identified in the cited databases. After reviewing the titles, 95 papers were selected, of which 23 were duplicates, resulting in 72 references that were then analyzed by title and abstract. Of these, 26 were eliminated as they did not line up with the objective of the research. Next, the full text of the resulting 46 papers were analyzed. Nine of these papers were eliminated as they were opinion studies, 33 were research projects or descriptive studies and did not provide comparative results, and four addressed topics different from the research. Finally, six studies were included in the review (Figure 1).

### Analysis of the Results

A narrative synthesis and vote count was conducted for the final six studies included in the review, grouping the results according to the outcome variable analyzed. Each study was quantified according to the direction of their results. Studies were considered “positive” if found a statistically significant improvement in the variable analyzed with the use of drones versus a traditional approach; “negative” if found statistically significant results in the group that attends the health emergency through traditional health intervention; and finally “null” when finding no statistically significant differences between the use of drones and the traditional approach.

## Results

All the studies included in the review presented acceptable values of robustness and quality in their methodology and results, as shown in Table 2 and Table 3. The experimental studies included^11,23^ in the review were of moderate-high quality. The main limitation found in these studies was the absence of a blinding process for participants and investigators. As main strengths of the research, the high applicability to the clinic and the randomization process of the participants was highlighted. The quasi-experimental studies^21,22,24,25^ showed an acceptable quality value, finding the main limitations in the blinding process, as did the experimental studies and the use of convenience samples.

Table 4 describes the characteristics of the included studies. The main applications of drones for health care were as follows.

**Table 4.** Characteristics of Selected Studies

Study	Type of Study	Principal Results
**Jain, et al**^11^	Randomized Controlled Trial	In day and night conditions, the time to triage was significantly longer for the group using the drones (8.86 vs 5.24; P = .002 and 11.03 vs 7.54 min; P = .002). No significant differences were found in triage outcome or in the order of priority set (P >.05).
**Claesson, et al**^21^	Quasi-Experimental Study	A total of 20 searches were conducted in each group. The intervention group covered significantly more surface area than the control group (4590 vs 2600m^2^; P <.001). The mean time to dummy contact was significantly shorter for the intervention group (0.47 vs 4.34 min; P <.001).
**Karaka, et al**^22^	Quasi-Experimental Study	A total of 20 searches were performed. The intervention group covered significantly more surface area (228613 vs 88322m^2^; P <.01) and took significantly less time to reach the dummy (8.9 vs 57.3 min; P <.01).
**Pardo-Ríos, et al**^23^	Randomized Controlled Trial	The mean distance travelled was significantly lower for the drone group (920 vs 1091.11; P <.0031) and the percentage of victims found was significantly higher for the drone group (92 vs 66.7% of victims; P <.0001). No statistically significant differences were found for the quality of triage (94 for control group vs 88.4% for intervention group; P <.4719), for the performance of airway opening maneuver (62.5 for control group vs 79.2% for intervention group; P <.3408), nor for the performance of hemorrhagic compression (41.66 for control group vs 8.3% for intervention group; P <.1573).
**Ochieng, et al**^24^	Quasi-Experimental Study	The average cost per sample for motorbikes was $24.06 while for drones it was $27.42. At distances of less than 30km, motorbikes dominate delivery times and costs; however, drones become more cost-effective when extended the distance to over 75km.
**Haidari, et al**^25^	Quasi-Experimental Study	For distances <75km, there is an improvement in availability (100 vs 97%) and a reduction in dose cost (0.22 vs $0.31 per dose). The cost reduction is more (0.17 vs $0.31 per dose) for greater distances.

### Accident Victim Location

Three studies^21–23^ evaluated the time taken to access the victim and the area travelled for tracking in searching for victims. For the first variable, these studies found that the time needed to reach the victim was significantly shorter for the group that used drones compared to the group that used traditional ground transport. Regarding the area covered, drones covered significantly more area than other traditional ground vehicle tracking methods.

### Triage Performance

Two studies^11,23^ evaluated the effectiveness of drones in performing casualty triage. In the research by Jain, et al,^11^ a statistical difference in time to complete triage was observed, being significantly higher in the group using drones than in the standard practice group. However, no differences were found between groups for casualty evacuation, order, and accuracy. These results were similar to those found in Pardo-Ríos, et al^23^ with no statistically significant differences found for the quality of triage, the performance of the airway opening maneuver, or compressions versus bleeding when necessary.

### Cost Reduction in Specimen Transport

Two studies^24,25^ conducted a cost-effectiveness analysis of the use of drones to improve the availability of vaccines and the transport of biological samples (in routine and emergency public health conditions based on the Ebola virus epidemic) between two sites. The studies were carried out in African countries in areas often constrained by poor road conditions, difficult geographical terrain, and insecurity. Both studies found that drones were significantly more effective at distances greater than 75 kilometers. For shorter distances, Ochieng, et al^24^ found that transport with a motorbike was more economical than transport with a drone.

## Discussion

The objective of this review was to evaluate the available evidence on the possibilities of using drones in emergency health care, finding them to be a novel and beneficial element in patient care.

Drones provided benefits over traditional care in addressing health emergencies, with study results centered on improved care by health care workers. However, the benefits go beyond improved care. Through interviews with doctors attending disasters, Hart, et al^26^ found that the images provided by the drones can be very useful in a real scenario for carrying out a preliminary triage, having knowledge of the scenario prior to arrival, and determining the needs that will be required to attend to the disaster.

Research by Sanfridsson, et al^27^ showed that it is beneficial in a simulated cardiac arrest situation where a drone is used to communicate with emergency service and simultaneously provide an automated external defibrillator/AED. In this situation, and when two observers witness the arrest, the degree of calmness is greater and the time without contact with the victim is representatively reduced compared to a situation with a single observer and where only telephone contact with the emergency service is made.

A variable analyzed in this review was the surface area covered by the drone for victim location, finding that drones offer significant benefits over a traditional search process, a result similar to that reported by Pulver, et al^28^ who found that drones covered 43% more surface area than the ground tracking group; however, statistical differences between the two groups were not evaluated. The ideal option would be to have drone networks that could reach an accident in less than three minutes, or even a network capable of covering 90% of the population in less than one minute, providing early health care.^28,29^

Another of the study variables was the effectiveness of drones in locating victims. Sibley, et al^12^ described that over 75% of victims were correctly located using drones; their greater effectiveness compared to other location methods cannot be confirmed, but they can be used as a complementary element or when there are risk factors for health teams. On the other hand, these authors have shown that younger people classify victims better,^12^ perhaps due to their greater confidence in new technologies.^5^

Drones can help in the search for survivors, gather information on the number of patients in need of care in disaster situations, assess injuries related to chemical, biological, and nuclear weapons, assist in surgical procedures being performed in difficult environments,^3^ and assess the general condition of victims, helping to determine their degree of consciousness or assessing the presence of respiratory movements.^3,30^

Drones provide potential benefits in health care; however, world-wide regulations should be developed for their correct use, taking into account the locations and situations where their use would be permitted by carrying out a risk-benefit analysis.^4,5^ Among the regulations determined in some countries are that they must be operated by a certified pilot, during daylight hours, with the drone in line of sight, and flight only in controlled airspace.^31^ It would also be necessary to control the drone’s intrinsic factors, such as flight speed, the payload it can carry, the characteristics and sensors that a medical drone must have, as well as extrinsic factors such as the weather conditions in which it can fly.^17,32–34^

Bhatt K, et al^35^ allude to ethical issues such as respect for confidentiality that may be breached if outsiders observe patient data or the delivery of medication. They also refer to the possibility of technical failure, the risk of collision, weather conditions, or the temperature difference that may alter the normal functioning of the device. Although as previously described, several studies have evaluated the feasibility of transporting biological material showing promising results that may improve the availability of samples, mainly in areas of difficult access, and optimize the cost of transporting them.^24,25^

## Limitations

Despite the potential advantages found in the review, the available evidence is limited because the technology underpinning drone flight and the functionalities of drones are constantly evolving. Hence, their characteristics can be improved to make them more functional in various critical situations and their benefits are expected to be greater. Another potential limitation is the lack of statistical significance in some of the outcome variables studied, as well as the heterogeneity of the results due to the different methodologies used.

## Conclusion

Drones can be considered a complementary element to traditional care when attending health emergencies, significantly improving the distance travelled to locate accident victims, providing the possibility of performing triage prior to the arrival of the health care units, and improving the time and quality of the care provided.
